# Heterotopic Ossification of Brachialis Muscle

**DOI:** 10.1100/tsw.2005.104

**Published:** 2005-09-29

**Authors:** Jacob George

**Affiliations:** Department of Physical Medicine and Rehabilitation, Christian Medical College, Vellore, Tamil Nadu, India

## Abstract

A 13-year-old girl with seizure disorder presented with 90º fixed flexion deformity of right elbow. She had history of encephalitis, 2 years ago, from which she recovered completely except for the deformity of the elbow. Plain X-ray revealed extensive ossification of the brachialis muscle from its origin at the lower anterior aspect of the humerus to its insertion at the coronoid process of the ulna. The alkaline phosphatase value was 500 IU. The middle segment of the ossified mass was surgically excised. The mobility of the elbow was restored and she achieved a range of movement between 45–120º.

